# A Systematic Scoping Review of the Resilience Intervention Literature for Indigenous Adolescents in CANZUS Nations

**DOI:** 10.3389/fpubh.2019.00351

**Published:** 2020-01-10

**Authors:** Crystal Sky Jongen, Janya McCalman, Roxanne Gwendolyn Bainbridge

**Affiliations:** Centre for Indigenous Health Equity Research, School of Health, Medicine and Applied Sciences, Central Queensland University, Rockhampton, QLD, Australia

**Keywords:** indigenous adolescents, resilience, community capacity, intervention research, health and well-being

## Abstract

**Background:** The concept of resilience offers a strengths-based framework for interventions to enhance Indigenous adolescent social and emotional well-being. Resilience interventions in or with schools encompass individual, social, and environmental factors that encourage health-promoting behaviors and assist adolescents in navigating toward resources that can sustain their health and well-being in times of adversity. This scoping review examined the literature on resilience-enhancing interventions for Indigenous adolescent students in Canada, Australia, New Zealand, and the United States (CANZUS nations). Intervention strategies, adherence to theoretical constructs, and outcomes were analyzed.

**Methods:** A systematic search was conducted of intervention studies aimed at improving Indigenous adolescent resilience and published in CANZUS nations between January 1990 and May 2016. Eleven peer-reviewed databases and 11 websites and clearing houses were searched for relevant studies. Following double-blinded screening, a total of 16 intervention papers were included for analysis. Study characteristics were identified and study quality was assessed using appropriate assessment tools.

**Results:** Twelve interventions (75%) were delivered in school settings and four (25%) were community-based, conducted in partnership with schools. Seven publications (44%) reported interventions focused exclusively on fostering individual resilience. Another seven (44%) included components that aimed to build staff, school, and/or community capacity to support adolescent resilience, and two (12.5%) had community/school capacity-building as the primary focus. Culturally based approaches to enhancing resilience were evident in most studies (81%). The publications documented the use of a range of program models, processes, and activities aligned with resilience theory. Positive outcomes were reported for improved individual assets (e.g., strengthened self-esteem and Indigenous identity), environmental resources (e.g., increased peer support and social/community connection), and increased community capacity (e.g., increased youth training and leadership opportunities). On average, study quality was assessed as moderate to high. The strongest evidence of intervention effectiveness was for improvements in mental health symptoms and outcomes.

**Conclusion:** Interventions indicated strong alignment with ecological and culturally based resilience theories and models. While the results of the studies indicate some positive impacts on the resilience of Indigenous adolescents, future evaluations should aim to ensure high study quality and focus on measuring strengths-based resilience outcomes.

## Introduction

Resilience is recognized as an essential process for maintaining and promoting child and adolescent mental health and protecting against potential threats to health and well-being throughout life transitions (1). “In the context of exposure to significant adversity, resilience is both the capacity of individuals to navigate their way to the psychological, social, cultural, and physical resources that sustain their well-being and their capacity individually and collectively to negotiate for these resources to be provided and experienced in culturally meaningful ways” (p. 127) (2). Resilience theory is increasingly used to study and understand the factors and processes that enable adolescents to negotiate and shape their environments, overcome adversity, and respond to life's challenges in healthy ways (3, 4).

Adolescence, defined as ages 10–19 (5), is a time of immense biological, physical, emotional, psychological, and social developmental changes (6–8). These changes drive major behavioral transformations that can both support positive development and lead to significant challenges (8). Adolescence is a period of heightened stress and turmoil (8), with difficult experiences, such as the transition from primary school to secondary school often having a negative impact on adolescent self-esteem and mental health (9). Mental health concerns, behavioral problems and substance use are major health problems facing adolescents across the globe (5, 10), with as many as 50% of adult mental health disorders beginning in adolescence (11). However, despite the challenges that often come with adolescence, there is substantial coping, adaptation, and resilience, even among those who have experienced significant adversity (12).

While there is evidence that different people exhibit different levels of resilience (13–15), we also know that resilience can be influenced and nurtured, and the earlier in life, the better (16, 17). Adolescence, as a transition period, can be “leveraged to encourage positive development trajectories” (p. 47) (8) through interventions to support the resilience of adolescents, such as peer interventions, individual and group psychological interventions, and school- and community-based programs (12). The promotion of resilience in adolescence is associated with lifelong benefits including improved participation in school and employment, increased pro-social outcomes, and possibly as an equalizer of socioeconomic inequalities (1). These benefits suggest that resilience-promoting interventions offer promise in meeting the needs of adolescents who experience limited opportunities to access health-sustaining resources in their everyday lives.

Indigenous adolescents in Canada, Australia, New Zealand, and the United States (CANZUS nations) experience higher rates of psychological distress, depression, and anxiety, as well as higher rates of self-harm, suicidal ideation, and suicide than non-Indigenous adolescents in all four countries (18–24). Research studies show that various socioeconomic risk factors and sociocultural determinants play a significant role in contributing to the inequalities in mental health and social and emotional well-being outcomes experienced by Indigenous adolescents (19, 20, 25).

Resilience-focused intervention research offers an appropriate strengths-based approach to enhancing the protective and promotive factors for Indigenous mental health (3). However, it is essential that the theoretical depth and complexity of resilience within particular contexts and for particular populations is understood in the design and selection of interventions (26, 27). To be effective, it is important that resilience interventions incorporate culturally appropriate approaches and are sensitive to the contexts of students' lives. These considerations are particularly important in culturally diverse groups of adolescents for whom mainstream societal norms and school culture are not a natural fit (28). Current evidence suggests that interventions ideally address key environmental factors rather than simply teaching adolescents to adapt to environments that do not always support their resilience (28).

Despite the promise of resilience intervention research for supporting Indigenous adolescent well-being, to date, there has been no systematic review of evaluated interventions to enhance Indigenous adolescent resilience. This scoping review was conducted to inform a school-based resilience-promoting intervention for Indigenous adolescents in Australia, including the core components needed for effective, relevant, and culturally safe interventions to improve Indigenous adolescent resilience in school settings. In the review, we identified the characteristics of interventions, the strategies used to enhance Indigenous adolescent resilience, and the theoretical consistency of these strategies, as well as the reported impacts on individual, community, and school resilience-related outcomes.

## Background

### Resilience: A Brief Overview of the Theory and Construct

The concept of resilience emerged from research about the impacts of adversity on mental health and development. Early pioneers in the field noticed that some individuals and families appeared to adapt more rapidly or recover better from adversity than others (29–33). These early researchers proposed that positive adaptation in the face of adversity was the product of a range of internal and external positive psychosocial resources (34). Since these initial conceptualizations, the field of resilience research has matured through four waves of research developing more sophisticated theories and understandings of resilience and their application (35, 36).

A major accomplishment of resilience research has been the identification of a consistent range of promotive and protective factors that support resilience (30, 36) on multiple levels, including individual, family, community, and society (13, 37–39). These factors can be conceptualized as assets or resources, with assets being individual-level protective and promotive factors and resources being contextual and environmental influences (3, 37, 40). While not exhaustive, Table 1 presents a large sample of some of the most common resilience-promoting factors identified across the literature (9, 30, 35, 36, 38, 39, 41, 42). Yet, despite the expansive and well-developed literature on the topic, “there is no universally accepted way of defining, quantifying, or measuring resilience” (p. 994) (42, 43). Resilience has been conceptualized as a trait, a process, or an outcome, or as a broad concept encompassing all of these (30).

**Table 1:** Resilience-promoting factors.

**Individual assets**	**Environmental resources**
• Self-esteem and confidence • Self-efficacy • Emotion regulation skills • Stress tolerance • Problem-solving skills • Information processing • Planning and decision-making skills • Intelligence • Educational abilities • Reflectiveness and personal awareness • Agreeable personality • Self-worth • Verbal communication skills • Social skills • Empathy • Hope • Faith • Optimism • Sense of direction or purpose • Belief life has meaning • Positive outlook on life • Educational aspiration and school commitment • Achievement motivation • Creativity • Humor • Religious affiliation and participation	**Family Factors** • Secure attachment relationships with parents • Authoritative parenting style • Caregivers who model competent behavior • Parental support of education • Parental supervision • Interpersonal warmth • Family cohesion • Emotional security and belonging • Marital harmony • Socioeconomic advantages • Religious/faith affiliation and participation • Strong relationships with extended family and kinship networks **Peer and Community Factors** • Friendships and romantic attachments with prosocial, well-regulated, supportive peers • Connections to stable, reliable, and supportive adult role models • Positive social support networks • Community well-being, stability, and cohesiveness • Opportunities for belonging and meaningful involvement in prosocial school, sports, religious, and community activities • Opportunities to engage in socially valued roles and activities that allow for responsibility, manageable challenge, and the opportunity to develop competencies and experience success • Opportunities for learning and mastery • Authoritative school and teacher styles, positive school climate and bonding to school **Culture/Ethnic Identity** • Strong, positive ethnic/cultural identity • Identification with traditional beliefs and values • Participation in traditional practices • Racial/ethnic socialization • Connection with members of one's cultural or social group

### Resilience in Cultural Context

Resilience, as a concept and process, is culturally, historically, and temporally embedded (4, 28). To be relevant in diverse settings across the world, it is essential that understandings of resilience and interventions aiming to enhance resilience are culturally and contextually embedded (28, 44). One major critique of early resilience research was that it did not generally address the implications of context and cultural variations in risk and resilience (13). However, there has been substantial research on the role of culture in resilience in the last two decades (13). Studies of resilience among youth across different cultures and contexts have found that there are global factors that contribute to young people's resilience, as well as culturally and contextually specific factors (45), including for Indigenous adolescents from CANZUS nations (46, 47).

A recent systematic review found that the most common factors associated with the mental health of Indigenous children living in high-income countries were the universal psychosocial protective factors of positive family and peer relationships, high self-esteem, and optimism (48). Research with diverse groups of Indigenous adolescents consistently demonstrates the central role that positive connections to a supportive family, friends, and the community play as resilience-promoting factors in Canada (49, 50), Australia (51–53), New Zealand (54, 55), and the United States (56, 57). Evidence suggests that such environmental resilience-enhancing resources are particularly important for Indigenous adolescents and that they have a greater impact on their resilience than do individual factors (56). While studies from Australia have found that higher self-esteem, self-regulation, self-respect, and positive future orientation are positively associated with Indigenous youth resilience (51, 52), and in the United States, self-efficacy, current or future aspirations, positive self-image, and personal wellness were all identified as resilience-enhancing protective factors (57), individual promotive factors are not as frequently identified in the Indigenous literature as environmental protective factors. One systematic review of culturally specific risk and protective factors for American Indian (AI) and Alaska Native (AN) youth well-being in the United States, for example, found that 59% of the identified factors had a relational aspect. In contrast, only 13% of risk and protective factors found were at the individual level (56).

Research into resilience-enhancing factors for Indigenous adolescents across the CANZUS nations also highlights the importance of cultural connection and expression for these populations. Studies on protective factors for AI/AN youth mental health and well-being have identified the key protective role of connectedness to culture (56, 57). One systematic review found that strong levels of ethnic identity or enculturation protect against substance abuse, mental distress, and suicidality and predict prosocial outcomes, that engagement in traditional spiritual practices and activities is a protective factor against substance abuse, and that commitment to cultural spirituality is protective against suicidal behavior (56). Qualitative research into promotive and protective factors for Inuit youth in Canada found that engagement in cultural activities like hunting, camping, and fishing (49) and connecting to cultural practices, traditions, knowledge, and Indigenous identity through being on the land (50) supported youth well-being and resilience. Qualitative research from New Zealand also found that a strong, positive Maori identity supported adolescent resilience at high school and produced a positive sense of belonging, community, and place and feelings of pride (54). Research from Canada and New Zealand also highlights the fact that, for many Indigenous adolescents, relationships with family and friends as well as a sense of collective well-being and responsibility are experienced as important to cultural connection and expression (50, 55).

However, there is mixed evidence regarding the role that culture plays in Indigenous youth resilience (48, 51, 56). For example, in Australia, qualitative research on resilience and well-being among Aboriginal young people indicated that strong cultural connection, cultural identity, and knowledge of cultural heritage and practices are considered to be an important source of resilience and well-being (52, 58). On the other hand, the results of a large survey found that connection to culture, measured by self-reported knowledge of culture, was not significantly associated with positive psychosocial functioning (51). An international systematic review also found evidence to be inconsistent regarding the impact of identification with culture among Indigenous young people. Fifty percent of studies reviewed found a significant association between identification with their own Indigenous culture and better mental health outcomes. However, some studies have found an association between cultural identification and poor mental health (48).

Given the centrality of connection to family, friends, community, and culture for Indigenous adolescent resilience, a social-ecological model of resilience that emphasizes the role of social and physical ecologies in positive developmental outcomes (59) is critical for research in this population. Within such a social-ecological model, resilience is considered to be determined by interactions between an individual and the systems in their broader environment (38), including families, peer groups, schools, cultures, and communities (30).

Such a social-ecological model can be used to inform interventions focused on developing resources across multiple levels, not only individual strengths (37). Principles developed to guide efforts in implementing resilience interventions suggest that interventions: (1) have a strong base in theory and inclusion of factors in one's environment that affect resilience; (2) have a foundation in research specific to target groups; (3) focus on the promotion of adolescents' adaptation and development rather than only on reduction of negative outcomes and on building on strengths and resources rather than only on reduction of risk/vulnerability factors; (4) target prominent protective and risk processes that play out across multiple levels of influence from individual, family, and community; and (5) consider context as a key driver (27). For Indigenous adolescents, resilience can be built through processes that teach or enhance resilience-promoting factors on multiple levels including the individual, social (family and peer network), and societal (school environment or community) levels (42) as well as through strengthening connection to culture and cultural identity (60).

## The Scoping Review

While there is a growing body of research on intervention approaches toward enhancing resilience for diverse adolescent populations (61), there is a lack of research focused on interventions to enhance Indigenous adolescent resilience. This scoping review evaluates the existing evidence base on interventions to promote the resilience of Indigenous adolescents in the CANZUS nations. Our approach is informed by a social-ecological perspective that emphasizes the interdependency between individuals and social systems (39, 62, 63). We consider resilience to be about both individual attributes or assets and external social, systemic, and physical resources (27, 64). This scoping review included interventions that supported adolescents experiencing adversity to strengthen their capacities for successful adaptation through enhancing competencies and resources at individual, family, community, and school system levels.

Scoping reviews are a knowledge synthesis method that can be undertaken to summarize and disseminate research findings, identify research gaps, and make recommendations for future research (65). Scoping reviews frequently address broader research questions than do typical systematic reviews and are therefore more suitable for topics where the literature has not been comprehensively reviewed or those that are complex and heterogeneous in nature (66). Whereas, traditional systematic reviews typically focus on a very specific, well-defined research question and only include studies of certain designs and quality, scoping reviews include studies of diverse designs, irrespective of their quality (65). They aim to map the key concepts underpinning a research area and address questions beyond those related to intervention effectiveness (66). They are particularly useful in emerging fields where a lack of randomized controlled trials makes systematic reviews of effectiveness unfeasible (67). Given the breadth and complexity of this research field, a scoping review methodology was chosen as the most appropriate approach.

## Methods

A systematic search was conducted to identify studies that aimed to either improve or measure the resilience of Indigenous adolescents in CANZUS nations. Included studies comprised intervention evaluations and indicator or correlation studies reporting the development, testing, or utilization of instruments used to measure resilience in Indigenous adolescents. This scoping review reports on the intervention strategies and outcomes reported across intervention studies. A separate review by the same authors examines the literature on instruments used to measure constructs of resilience in Indigenous adolescents (68).

As a first step in the review process, a study protocol was developed to define the research objectives and methods in line with the Joanna Briggs Institute (JBI) Reviewers Manual on scoping review methodology (69). This protocol outlined the proposed questions, inclusion and exclusion criteria, search strategy, and data extraction process for the review. The protocol was revised and approved by all authors.

The review aimed to answer the following research questions. (1) What are the characteristics of the strategies utilized in evaluated interventions that aim to increase the resilience of Indigenous adolescent students? (2) What are the reported outcomes of intervention evaluations, and where does the best evidence of intervention effectiveness lie? (3) How do the evaluated interventions adhere to the theoretical foundations of resilience consistent with a social-ecological perspective?

During the data-extraction process, the researchers sometimes struggled to distinguish between interventions focused on improving mental health and those aimed at building or enhancing resilience. The absence of or lower incidence of mental health issues has commonly been used by resilience researchers as a measurement of resilience along with positive outcomes and achievements (13, 42); however, this approach of defining resilience through the absence of mental health issues has received strong criticism (30) since resilience is not necessarily associated with the presence or absence of a mental health diagnosis (42). Resilience implies a focus on positive outcomes (27). The authors, therefore, excluded studies that did not incorporate at least one resilience-focused or strengths-based outcome.

## Inclusion/Exclusion Criteria

Studies in this review included peer-reviewed and gray literature published in English from January 1st, 1990 to May 31st, 2016 inclusive. The start date of the review was taken from 1990 to coincide with the third wave of resilience studies that focused on promoting resilience through intervention (35). The criteria for inclusion were studies:

- from Australia, Canada, New Zealand, or the United States;
- targeting Indigenous adolescent students between the ages of 10 and 19 years;
- evaluating an intervention conducted in school settings or in partnership with schools that was designed to improve the resilience of Indigenous students through enhancing individual and/or environmental competencies and resources at student, school, community, and/or staff levels; and
- measuring at least one resilience construct as an intervention outcome.

## Search Strategy

**Figure 1:**
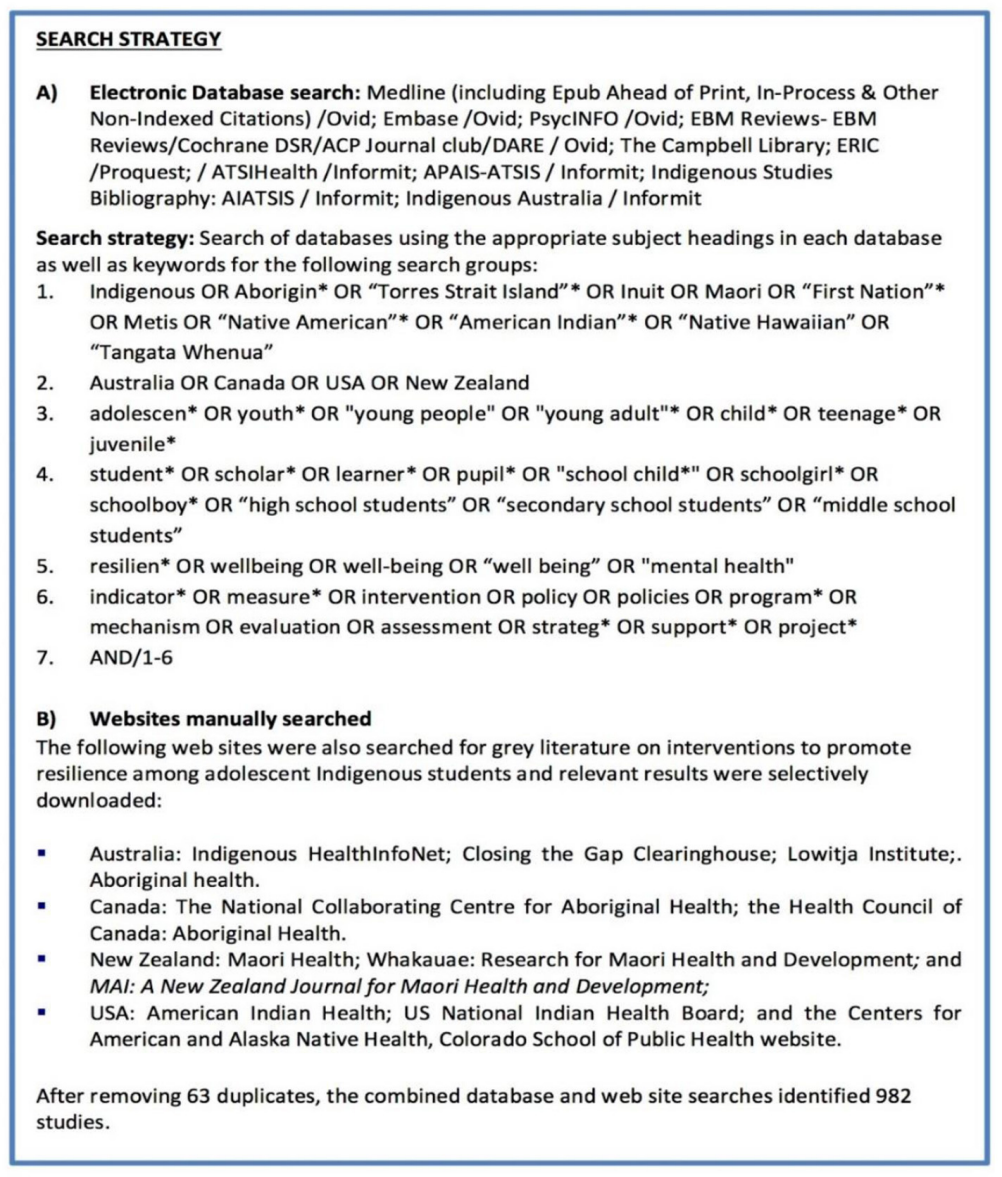
Search strategy.

The search strategy comprised four steps (see Figure 1).

**Step 1**: A librarian (MK), expert in systematic reviews of Indigenous health-related literature, searched 11 relevant electronic databases and identified 969 references, excluding duplicates.

**Step 2**: Gray literature from 11 clearinghouses and websites of relevant organizations in each of the four countries was searched by a research assistant (KR) overseen by one author (JM). Thirteen more publications were identified.

**Step 3**: The 982 identified references were imported into Endnote, and their abstracts were manually examined; 33 studies were identified as potentially meeting the inclusion criteria.

**Figure 2:**
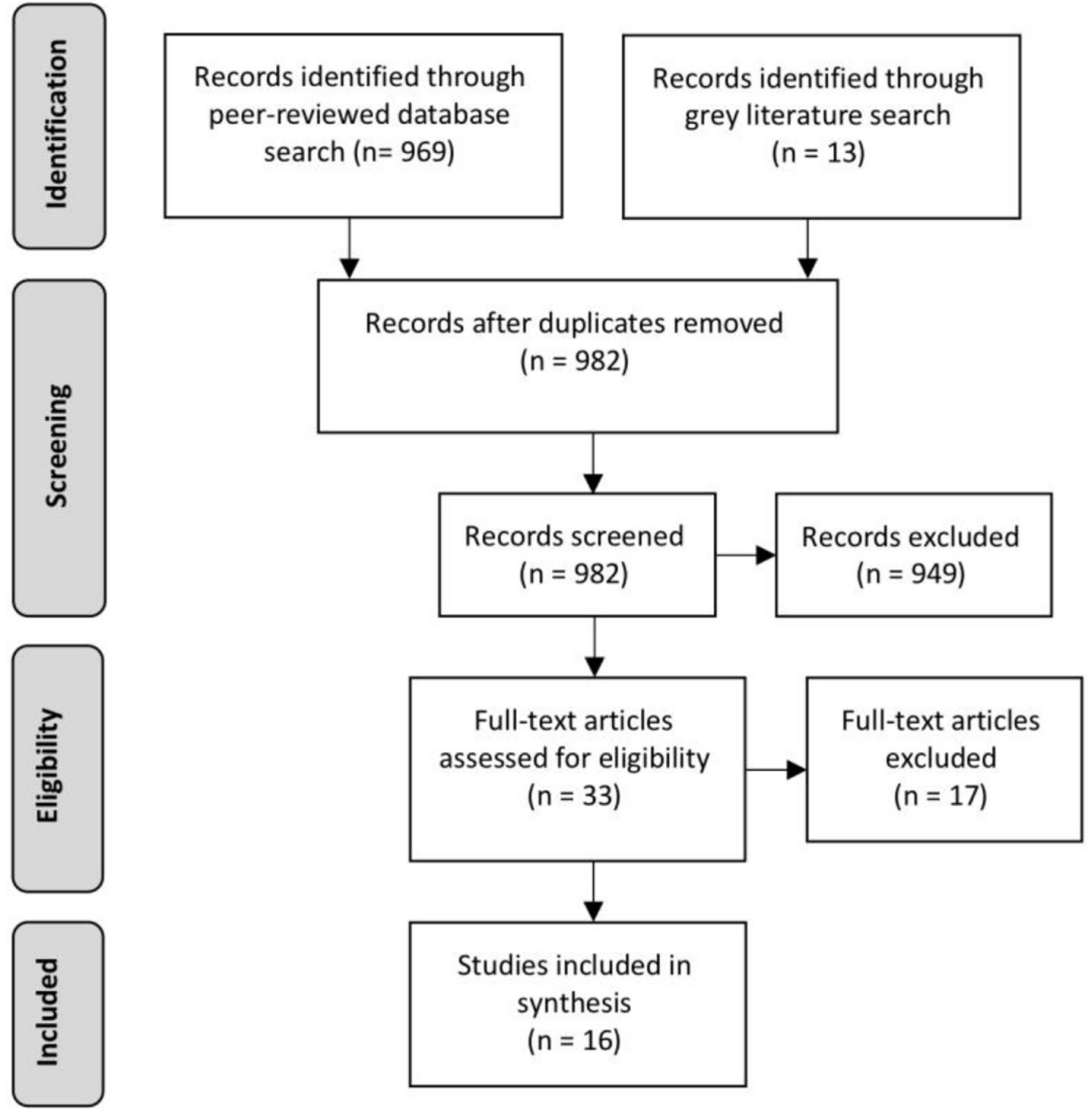
Prisma flow chart.

**Step 4**: The full text of the 33 articles was examined. Eight descriptive studies, four correlation or indicator studies, and five intervention studies that were not conducted in school settings or in partnership with schools were excluded. A total of 16 intervention studies were included for analysis. See Figure 2 for a flow chart of the PRISMA search strategy (70).

## Identification, Screening, and Inclusion of Publications

The results of the searches of both peer-reviewed and gray literature were imported into the bibliographic citation management software, Endnote X7, with duplicates removed. Titles and abstracts of publications were screened by one author (CJ); those that did not meet the inclusion criteria were excluded. The full texts of the remaining publications were retrieved and screened by blinded reviewers (RB, JM). Inconsistencies in reviewer assessments were resolved by consensus.

## Data Extraction and Analysis

Data extracted from the full texts of the studies included: publication authorship, year, and type; country and target group; sample size and intervention setting; intervention type and study design; outcome measures; reported outcomes; study quality (see Supplementary Table 1 for all extracted data). The quality of the included quantitative studies was assessed using the Effective Public Health Practice Project quality assessment tool (71). Qualitative studies were assessed using the Critical Appraisal Skills Programme quality assessment tool (72). Assessment of intervention effectiveness was determined based on the evidence typology outlined by Petticrew and Roberts (73). The reviewed intervention studies were analyzed to identify key intervention characteristics in terms of the interventions implemented and the outcomes reported.

## Results

A total of 16 intervention studies that evaluated programs designed to increase the resilience of Indigenous adolescents were identified. Six studies came from Australia (74–79), six from the U.S.A. (80–85), three from New Zealand (86–88), and one from Canada (89). Studies across the four countries varied in their approach. Seven of the 16 (44%) studies reported interventions focused on improving resilience at the individual level (75, 76, 79, 83, 85–87). Another seven studies (44%) included a staff training component to increase school or community capacity (77, 80–82, 84, 88, 89). One study aimed to simultaneously address both individual student resilience and community capacity to support improved resilience among adolescents (74), while another study reported an intervention that solely addressed community and school capacity to improve adolescent resilience (78). Of the 16 studies reviewed, 13 (81%) included activities focused on cultural engagement or participation (74–77, 80–86, 88, 89).

The interventions targeted a wide age range of young people, from grade 1 (age 5) to 26 years of age, with the majority targeting adolescents in their secondary school years (see Supplementary Table 1 for target groups by study). Diverse Indigenous populations were represented including: First Nations/Native American (80–83, 85) and Alaskan Native (84), Australian Aboriginal and Torres Strait Islander (74–79), Maori (86–88), and Canadian First Nations/Aboriginal (89) peoples. Twelve interventions (75%) were delivered in school settings (75, 76, 78–83, 85, 87–89); four (25%) were community-based, conducted in partnership with schools (74, 77, 84, 86). Nine of the included studies (56%) were published between 2010 and 2015, five studies (31%) were published between 2005 and 2010, and two were published between 1995 and 2005 (12.5%).

## Intervention Strategies

A range of program models, aims, and activities were identified across the evaluated interventions. We use the term “program aims” to describe the kinds of resilience skills, capacities, and resources the interventions aimed to enhance. “Program models” refers to previously established programs, frameworks, and approaches that were used to guide intervention development and implementation. “Program activities” include the suite of program components utilized in interventions.

## Program Aims

The program aims explicitly identified across the reviewed studies are outlined in Table 2. The strengthening of Indigenous identity was a program aim that was incorporated in some form in the majority of interventions; however, some made this process more explicit or focused more on the strengthening of Indigenous identity than others (74, 75, 83).

**Table 2:** Program aims.

**Program aims**	**Studies addressing aims**
**INDIVIDUAL ASSETS PROGRAMS AIMED TO ENHANCE**	
- Problem-solving skills (50%)	(74–76, 82, 84, 87–89)
- Processes and skills for understanding and managing difficult emotions (50%)	(74, 79, 82, 84, 86–89)
- Social/emotional/life skills (44%)	(74, 75, 79, 81, 85, 87, 88)
- Leadership development (37%)	(74, 76, 77, 79, 81, 84)
- Self-esteem, confidence, and self-efficacy (31%)	(75, 82–85)
- Decision-making skills (19%)	(75, 76, 83)
- Goal-setting skills (19%)	(75, 82, 83)
- Communication skills (12%)	(76, 82)
**ENVIRONMENTAL RESOURCES PROGRAMS AIMED TO ENHANCE**	
- Social bonding with peers (31%)	(75, 76, 84, 85, 88)
- Connection with family, community, and culture (31%)	(74, 75, 79, 83, 84)
- Connection to positive role-models (19%)	(74, 79, 84)
- Increased community participation and engagement (12%)	(74, 75)
- Increased community capacity to improve indigenous adolescent resilience (12%)	(74, 78)

## Program Models

Diverse program models (previously established programs, frameworks, and therapeutic approaches) guided interventions, including both mainstream and Indigenous models. Cognitive Behavioral Therapy (CBT) featured in four interventions (84, 87–89), three of which included cultural enhancements (84, 88, 89). Other mainstream program models included life skills training (76, 82) and peer support/education models (76). Social Cognitive Theory, which aims to develop peer and community connection and draw on positive role models to promote resilience while also enhancing individual self efficacy, was identified as the foundation of interventions in two studies (82, 84). The Applied Humanism Model of caregiving was applied in one intervention as a framework for managing behavior while fostering student social and emotional development (81). The Circle Solutions Framework was used in another intervention to promote principles of inclusion, respect, safety, and positivity while teaching skills to foster resilience and well-being (75). Social Emotional Learning (SEL), a process for teaching the intrapersonal and interpersonal skills fundamental to personal resilience and healthy relationships (90, 91), was used as the basis for cooperative group processes to foster positive peer connection and support between participants (74, 75, 79, 85). A further two community-based, multi-site interventions identified the use of community development principles as the foundation of programs (74, 77). In those two studies, the focus was on collaborative, community-driven responses to increase adolescent resilience.

Several interventions were also informed by Indigenous-developed models. The Circle of Courage provided a strengths-based framework for two interventions for Native American adolescents (80, 85). It outlined the need to rebuild the “courage” of adolescents through (1) experiencing belonging in a supportive community, (2) meeting their needs for mastery, (3) involving them in determining their own future, and (4) encouraging their independence (80). Another intervention for Native American adolescents was based on the Cherokee self-reliance model (83). This model comprises three capabilities: being responsible (caring for oneself and others), being disciplined (setting and pursuing goals, taking initiative and risks, and making decisions), and being confident (having a sense of identity and self-worth, as well as strong cultural values and beliefs) (83). Lastly, one Australian study evaluated an intervention based on the Aboriginal-developed adult Family well-being empowerment program, adapted for use with Aboriginal and Torres Strait Islander children in school settings (79). The main aims of this adapted program were to build personal identity, help students recognize their future potential, and develop awareness of their place in the community and society (79).

## Program Activities

The studies reported the use of a wide range of program activities, including group workshops, cultural engagement and participation, education, training, mentoring, and community capacity building. While highly diverse, these program activities all aimed to increase student resilience. These program activities are outlined in Table 3.

**Table 3:** Program activities.

**Program activities**	**Studies utilizing activity**
**GROUP WORKSHOPS**	
- As part of multi-component intervention	(74, 76, 77, 80, 81, 84–86)
- As a sole intervention strategy delivered in schools	(75, 79, 82, 83, 88, 89)
**CULTURAL ENGAGEMENT OR PARTICIPATION**	
- Religious or traditional ceremonies and customs	(80, 81, 84, 85)
- Culturally grounded, enhanced, or tailored curriculum	(82, 83, 88, 89)
- A specific cultural program or class	(77, 81)
- Learning native languages	(81, 85)
- Attending cultural events or cultural excursions in the community	(75, 85)
- Outdoor/nature-based activities	(74, 77)
**Involvement of family and community**	
- Participation of community Elders and leaders in program delivery	(75, 81, 84)
- Making activities open for family and community	(74, 86)
- Building community contact/support for adolescents	(75, 85)
**Creative activities**	
- Art	(74, 76, 77, 81)
- Music	(74, 80, 81)
- Film and media	(76, 77)
- Dance	(85)
**EDUCATIONAL ACTIVITIES**	
- Education about risk factors	(74, 76, 83, 84)
- Mental health and suicide risk education	(82, 89)
- Social or life skills education	(76, 80–82, 85)
- Cognitive behavioral education	(87, 89)
- Academic support programs	(80, 81, 85)
- Technical studies and a learner driver's license program	(77)
**RECREATIONAL OR SPORTS-FOCUSED PROGRAM ACTIVITIES**	
- Physical fitness and nutrition programs	(80, 81, 85)
- Sport and recreation	(77, 84)
**PROGRAM ACTIVITIES TO IMPROVE THE PHYSICAL AND MENTAL HEALTH OF ADOLESCENTS**	
- Medical care	(76, 80, 81, 85)
- Mental health care, counseling, and case management	(77, 80, 81, 84–86)
- Staff smoking cessation program	(85)
**TRAINING, MENTORING, AND EMPLOYMENT OPPORTUNITIES**	
- Training for program staff	(74, 77, 80–82, 88, 89)
- Training for staff from other service providers, teachers and community members, teen advisors, and adolescents	(74, 84, 89)
- Mentoring or coaching	(74, 75, 80, 83)
- Skills training for adolescents	(76, 81)
- Suicide prevention/intervention training for adolescents	(82)
- Cultural competence training for staff	(81)
- Employment opportunities for adolescents and local community members	(74, 77)
**COMMUNITY CAPACITY BUILDING**	
- Delivery of training to community members, service providers, and adolescents, support and mentoring to youth workers in partner organizations, and employment training and mentoring of local Aboriginal staff	(74)
- Establishing learning communities between local Anangu educators, skilled linguists, community members, and trained Mind Matters mental health staff to develop local language around mental health and well-being	(78)

## Intervention Outcomes

The intervention evaluations reported a range of positive outcomes. Several of these maintained a strengths-based focus, while others, although potentially indicative of increased resilience, were framed from a deficit viewpoint. The most common outcome reported was improvement in the individual resilience assets of participating adolescents. Specifically, these were improvements in: coping skills and communication/conflict resolution skills (74, 75, 86, 88), self-esteem and/or confidence (74–76), acceptance of seeking support (76, 85, 88), leadership capacity, personal power and autonomy, and sense of purpose (74, 75), knowledge and awareness/understanding of alcohol, drugs, and suicide (74, 76), sense of Indigenous identity (74, 75), positive attitudes (75, 88), analytical and reflective skills and ability to set goals (79), self-reliance and improvements in overall health (83), and scores on pre-post measures of adjustment, interpersonal relationships, and adaptability (81). Improvements in resilience-promoting resources were also reported in the form of increased peer support/social inclusion and/or social connection/involvement (74, 75, 79, 85). Several positive but non-resilience-based outcomes (those couched in the language of deficit) were also reported, including those specific to improvements in mental health measures. These included: reduced substance use (76, 81, 83–86), decreased depression symptoms (87, 88), reduced anxiety for students with elevated anxiety (89), reduced feelings of hopelessness and decreased suicidality (82), reduced student self-harm (85), improved social and psychiatric functioning and reduced risk of clinically significant mental health concerns (86), and anecdotal evidence of reduced violence (78).

Diverse community- and school-level outcomes were also reported. Community-level outcomes included: increased community/staff knowledge, skills, and confidence (74, 76, 78), increased adolescent training and leadership opportunities (74, 77), and increased communication/coordination between stakeholders (74, 77). School-level outcomes included reduced behavioral incidents, increased student retention rates, increased academic proficiency (81, 85), decreased money spent on external mental health services (81), decreased teasing and bullying (79), and increased numbers of graduations (85). Osborn reported several positive outcomes for both school and community. These included the development of local language and understanding of mental health and well-being and the development of mental health and well-being-promotion resources in the local Indigenous language (78).

The interventions with the strongest evidence of effectiveness, those using randomized control trials or pre-post/multiple time series designs with moderate to strong study quality and reporting positive intervention impacts, generally measured changes in mental health symptoms (82, 86–89). Only one intervention using a pre-post design with a control group reported significant improvements using resilience-focused outcome measures (83). While positive outcomes were reported in the other studies, they were of inadequate quality to allow adequate assessment of intervention effectiveness.

## Study Methods and Quality

The reviewed studies used a diverse range of quantitative, qualitative, and mixed-methods study designs to evaluate interventions. Through our quality assessment, five studies were rated as strong in quality (74, 79, 82, 86, 89), five as moderate (77, 78, 83, 87, 88), and six as weak (75, 76, 80, 81, 84, 85). The majority of studies (9/16, 56%) employed quantitative study designs, with multiple time series designs (4/16 25%), randomized control trials (3/16 19%), and pre-post intervention designs (2/16 12%) being the most common. A further four studies employed mixed-methods designs, and two studies used qualitative designs.

A wide range of outcome measures were assessed by diverse measurement instruments across the included studies. Only three studies evaluated the same outcome measures using the same measurement instruments (80, 81, 85), although the interventions evaluated in these three studies were distinct. Of all the other measurement instruments employed, none were utilized in more than one study. However, some of the different measurement instruments used across studies assessed the same or similar constructs, such as substance use, depression, and anxiety. Supplementary Table 1 provides further details on the methods used in each study, their associated outcomes, and the study quality rating.

## Discussion

A small and diverse body of literature regarding interventions to enhance or improve Indigenous adolescent resilience in school settings or in partnership with schools was found. There was notable heterogeneity across the included studies, with the interventions employing a range of different program models, aims, and activities. Studies also utilized highly diverse evaluation methods and measurement tools to assess intervention impact, with varied study quality. Although this heterogeneity complicates the assessment of overall intervention impact and the evaluation of best-practice approaches, it is evident that the reviewed interventions produced many positive outcomes.

## Intervention Strategies and Their Theoretical Adherence

Some of the models utilized were primarily focused on enhancing personal attributes and internal resources to promote Indigenous adolescent resilience. Mainstream models, such as cognitive behavioral therapy (CBT) were used to teach adaptive thinking, problem solving, and coping styles. Life Skills Training models were used to increase adolescents' self-awareness, empathy, problem solving, communication, and interpersonal skills (92). The enhancement of individual assets is an important strategy that can be used in resilience-promoting interventions. However, in interventions for Indigenous adolescents, it is important that such individual-focused strategies are combined with strategies focused on enhancing promotive and protective environmental resources. Efforts to strengthen positive relationships with peers, family, schools, and communities are arguably more critical in Indigenous cultural contexts.

Given the central role of connection to family, peers, and community in supporting the resilience of Indigenous peoples (48–50, 52, 54–57), it is promising that a social-ecological approach was reflected in the program aims and models of the reviewed interventions. The interventions commonly aimed to enhance individual assets, for example, through teaching problem solving and social, emotional, or life skills or strengthening Indigenous identity, self-esteem or self-efficacy. But they also aimed to enhance resilience-promoting resources through strengthening social bonding with peers, fostering connection with family, community, and culture, and making use of role models.

Indigenous adolescent resilience intervention approaches also aimed to strengthen cultural identity and cultural connection (52, 60, 93–96). Given the positive impact strong cultural connection can have on Indigenous adolescent well-being and resilience (48–50, 52, 54–57), it is promising to see the utilization of Indigenous-developed models that are based in culturally grounded concepts of resilience, well-being, and positive development. It is also positive that several interventions using non-Indigenous models included cultural enhancements. Further research is needed into the role that culture plays in supporting the resilience of diverse Indigenous adolescent populations and how culture can be incorporated into the models utilized to inform resilience-enhancing intervention design and implementation.

## The Translation of Theory Into Intervention Activities

Our findings suggest that in line with social-ecological and cultural resilience models, interventions for enhancing Indigenous adolescent resilience ideally incorporate activities to enhance individual assets, community/social resources, and cultural connection and identity (3, 95). Intervention studies reported diverse intervention activities for building resilience-promoting assets and resources on the individual, peer, and community levels, particularly through group-based activities. Given the central role that peers and supportive friends play in protecting against diminished mental health and other problems and in building resilience for Indigenous adolescents (49, 97), group-based interventions are a promising approach for enhancing Indigenous adolescent resilience in school settings (74, 75, 79, 85). Group-based program activities also fostered a connection with caring adults, such as program facilitators, Elders, and other community members. Positive relationships with caring adults are a crucial psychosocial resilience-promoting resource for Indigenous adolescents (3, 42), as is connecting with community role models (60).

Many interventions also integrated program activities to enhance resilience through engagement in meaningful activities, such as engagement in traditional cultural practices and creative pursuits aimed to build both personal assets and community resources and Indigenous identity and cultural connectedness and to provide an opportunity to participate in socially valued roles. There is evidence that participation in group activities involving art, music, and sport, as well as participation in the creation of community projects and events, can enhance both individual and group well-being for adolescents (98–100). For example, visual arts and music programs have been used to improve the mental health and well-being of adolescents and both educate about resilience and increase overall resilience (101, 102). Such activities can provide the space for adolescents to initiate ideas, develop skills, and participate in decision making (98), and enhance personal identity and sense of self as well as building social networks and support (99). Hence, activities that provide opportunities for such meaningful participation and engagement and that can be implemented successfully in school settings appear to be an important strategy for enhancing Indigenous adolescent resilience.

Although there is some evidence to the contrary (103, 104), the international literature on resilience and well-being among Indigenous adolescents indicates that strong cultural identity as well as cultural affiliation and engagement, including native language competence and participation in cultural activities, has significant resilience-enhancing benefits (48–50, 52, 54–57, 104–108). Therefore, it was positive that many interventions encompassed activities that focused on enhancing cultural identity through engaging in and learning about cultural traditions and practices. Strengthening connections with family and community and participation in cultural activities were a common focus of intervention activities. There were however, only two studies (81, 85) that identified intervention components of teaching native languages. Given the resilience-enhancing benefits of native language competence (104–108), this activity merits further investment and exploration.

## Environmental Capacity-Building Approaches

Given the central role of environmental contexts in both the risk factors that can negatively impact resilience and the promotive and protective factors that enhance it (1), it was promising that the studies incorporated efforts to improve the resilience-enhancing capacity of schools and community environments. In school settings, environmental capacity-building occurred mostly through the provision of training to school staff (80–82, 88, 89). However, such training was generally not specified as a school capacity-building exercise, and examples of other initiatives to build school capacity were not provided. One study evaluated an Australian government school/community capacity-building intervention that involved schools, the community, and mental health services in developing local, culturally appropriate language, knowledge, and resources about mental health and well-being to enable educators to better address such issues in community and school contexts (78). This intervention reported outcomes, such as increased community/staff knowledge, skills, and confidence, the development of local language and understanding of mental health and well-being, and the development of health-promotion resources in the local Indigenous language (78). Such approaches to building school capacity through providing training to teachers to increase their knowledge about the psychosocial well-being and resilience needs of adolescents is a promising approach that warrants further research attention (109, 110). However, evaluation efforts also need to assess the impact of such approaches on individual students' resilience outcomes as well as the staff-, school-, and community-level outcomes.

Community capacity-building intervention approaches were also employed in several community-based interventions in the form of training and mentoring (74, 77, 84). One community-based intervention had community capacity-building as a core focus (74). While some interventions were conducted in schools and others in community settings, most interventions involved collaboration, partnerships, and participation across school and community boundaries. However, as found in previous resilience research (98), there was a lack of approaches that engaged the whole family. Families are a fundamental part of adolescents' environments and play a central role in supporting resilience (48–50, 52, 54–57). More cross-system, collaborative community capacity-building initiatives that involve family, such as those outlined by Blignault et al. (74), are needed (56).

## The Success of Interventions in Increasing Resilience

Despite study quality issues and heterogeneity in study methods and measures, there is good evidence that the interventions in the reviewed studies were successful in promoting the resilience of Indigenous adolescents. Most commonly reported were individual outcomes demonstrating improvements in individual resilience-promoting assets, including increased confidence and self-esteem, improved coping skills, and an increased sense of Indigenous identity.

Assessing changes at the community level can be more difficult than assessing individual-level outcomes (100), but the reviewed studies reported promising results of increased community capacity to improve adolescent resilience. There was also strong evidence of improved peer connection and support and increased community involvement/connection. Increased adolescent leadership and training opportunities, as well as increased knowledge, confidence, and skills among staff and community toward supporting adolescent resilience and well-being were also reported.

However, to test the effectiveness of interventions, it is important that appropriate study methods are used (73). The strongest evidence of intervention effectiveness demonstrated reductions in mental health symptoms. Only one study using an appropriate method and demonstrating moderate study quality assessed strengths-based resilience outcomes (83). While improvements in mental health and well-being as indicated by decreases in levels of depression and anxiety or suicidality can be seen as indicative of improved resilience, it is important that resilience-based research focuses on assessing positive outcomes (13, 27, 30, 42). To determine intervention effectiveness in increasing Indigenous adolescent resilience, there is a need for randomized control trials or cohort and quasi-experimental studies that utilize measurement instruments and outcome measures reflective of strengths-based resilience-promoting factors (68) that are relevant to the study aims.

## Limitations of the Literature and Implications for Research and Practice

Most studies reported positive outcomes, and many were assessed individually as being moderate to strong in terms of study quality. However, significant heterogeneity in terms of intervention design and implementation, as well as in evaluation methodology and assessment tools, makes comparison of intervention effectiveness and assessment of best practice approaches untenable in this review. For example, no one intervention was implemented or evaluated in more than one context. Therefore, there is no evidence to suggest that these interventions would be successful in other contexts or with other Indigenous adolescent populations. Comparison and/or replicability of intervention outcomes is further complicated by the paucity of studies that assessed the same outcome measures or assessed outcomes using the same measurement tools. A separate review of measurement tools to assess Indigenous adolescent resilience completed as part of this project similarly found that no one measurement instrument was used in more than one reviewed study (68).

The results of this scoping review suggest that a full systematic review or meta-analysis of intervention effectiveness would not be feasible due to significant heterogeneity in intervention designs and evaluation methods. Similar issues with heterogeneity have been found in other such reviews of complex issues related to health and well-being (111). In a recent systematic review of systematic reviews on mental health interventions for adolescents, for example, it was found that meta-analysis could not be conducted in most of the systematic reviews reviewed due to heterogeneity in the populations, interventions, and outcomes (112). To begin examining the true effectiveness of interventions to improve Indigenous adolescent resilience in school settings, there is a need to standardize interventions and outcomes (112) to allow comparison of equivalent studies with high-quality design. However, intervention heterogeneity will be an ongoing issue, particularly given the importance of tailoring interventions to suit local needs and contexts and of co-design and implementation with local communities. Future research developments need to strike a balance between these issues.

One potential way forward to create greater consistency in intervention approaches could be to build on the findings of this review as well as those of other relevant literature, e.g., (113), to establish a set of best-practice principles and approaches to inform intervention development. Interventions designed from such a set of best-practice principles and approaches could be implemented and evaluated in various school contexts, across a range of settings, using the same or comparable outcome measures and measurement tools. Using a randomized, multiple-time-series design is one potentially feasible way to better understand the impact and importance of different aspects of best-practice intervention approaches. This could be achieved by adding intervention components over time in a staged approach in individual schools, as well as through randomization and waitlisting of case/control schools [e.g., McCalman et al. (4)]. Such a research agenda is obviously ambitious and would require significant investments of time and resources. However, such an approach needs to be considered if we are serious about building a strong evidence base on what works best to improve Indigenous adolescent resilience.

## Limitations

Although the included publications were identified through a comprehensive search strategy that included electronic databases and websites/clearinghouses, it is possible that some relevant publications were not found. There is also a risk of publication bias, with studies that did not find positive results being less likely to be published. Although one study reporting negative outcomes was found and included in this review (80), the risk of publication bias is common for systematic reviews (114). Furthermore, many existing publications may not be available in key international databases (114). The authors of the review are based in Australia and have extensive knowledge and experience in Indigenous health equity research in the Australian context. Due to this direct knowledge and experience, several known databases specific to Australian Indigenous health research were searched. Similar Indigenous-specific databases from other included countries are unknown to the reviewers. This may have resulted in some bias toward Australian studies.

## Conclusion

The reviewed literature demonstrates that resilience-promoting interventions for Indigenous adolescents can take many forms and indeed need to be based on local needs, aspirations, and contexts. While various intervention models, processes, and activities were reported in the reviewed studies, they were generally aligned with ecologically based resilience theories and associated hypothesized change processes and included culturally based intervention models, processes, and activities to build cultural resilience in Indigenous adolescents. Many studies reported positive intervention outcomes at the individual level, as well as at the community and school levels. The strongest evidence of intervention effectiveness came from studies assessing intervention impact on mental health outcomes. While positive, outcomes, such as reduced mental health symptoms do not necessarily indicate enhanced resilience. Issues with the heterogeneity of intervention strategies, study designs, measurement instruments, and outcomes measured limit the ability to draw conclusions about the effectiveness of evidence-based, best-practice interventions for enhancing Indigenous adolescent resilience. To build a strong evidence base for what works in increasing Indigenous adolescent resilience, for whom, where, and why, investment is needed in coordinated, long-term research projects.

## Author Contributions

RB and JM designed the study protocol used for the search. CJ was responsible for the initial screening of search results and data extraction for included studies, and RB and JM completed the second screening. CJ was primarily responsible for writing the review draft, and RB contributed to the drafting of the review. RB and JM contributed to the feedback and edited the review. All authors approved the final version for submission and agree to be accountable for all aspects of this work.

### Conflict of Interest

The authors declare that the research was conducted in the absence of any commercial or financial relationships that could be construed as a potential conflict of interest.

## Supplementary Material

The data extraction table for all reviewed studies can be found in Supplementary Table 1.

## Abbreviations

CANZUS: Canada, Australia, New Zealand, and the United States
CASP: Critical Appraisal Skills Programme
CBT: Cognitive Behavioral Therapy
EPHPP: Effective Public Health Practice Project
JBI: Joanna Briggs Institute
PRISMA: Preferred Reporting Items for Systematic Reviews and Meta-Analyses
SEL: Social Emotional Learning.
