# Supplementary material for: A Systematic Scoping Review of the Resilience Intervention Literature for Indigenous Adolescents in CANZUS Nations
Source: Front Public Health. 2020 Jan 10;7:351. doi: 10.3389/fpubh.2019.00351 (PMC6967740; doi:10.3389/fpubh.2019.00351)
Supplement: Supplementary file 1 [file Table_1.DOCX]

| **a**  **1^st^ Author/**  **Publication Year**  **Publication Type** | **Intervention**  **type** | **Country developed/**  **Target**  **group** | **Sample Size** | **Study design** | **Outcome**  **Measures/**  **Measurement**  **Instruments** | **Outcomes** | **Study**  **Quality** |
| --- | --- | --- | --- | --- | --- | --- | --- |
| **Blignault (2015)**  **Journal paper** | A national program to improve the social and emotional wellbeing (SEWB) of Indigenous youth in remote and regional areas | Australia  Aboriginal and Torres Strait Islander youth aged 16-26 | 14 sites across Australia included in evaluation.  25 interviews were held with 6 national managers, 9 state/terriroty/regional managers, 10 Community Development Officers.  Interviews and focus groups were held with 27 community level informants, including program participants and their parents, Elders and community members. | Process and outcome evaluation though open inquiry and audit review, supplemented by five in-depth case studies.  Data collection methods included document analysis, in-depth semi-structured interviews and focus groups in the form of yarning cirlces´, and participant observation. | n/a | More established programs – with strong local partnerships and less staff turnover – made more progress.  Reports from program participants and parents described a range of personal outcomes including: increased self-esteem and ability to speak up; learning how to deal with painful emotions without resorting to alcohol and drugs; making friends; improved relationships; increased confidence at school and in life generally; and a new sense of Aboriginal identity.  Enhanced community capacity was also reported through increased knowledge, skills and confidence of the local Aboriginal staff. | Strong |
| **Clark (2014)**  **Journal paper** | Facilitated access to free counseling support | New Zealand  Culturally diverse youth | 581 culturally diverse youth aged 10–24 | Quasi-experimental pre-/post intervention design | **Outcome Measures:** Social and psychiatric functioning; mental health concerns; and, use and impact of drugs/alcohol.  **Measurement Instruments:** Strengths and Difficulties Questionnaire (SDQ), Substance Abuse Choices Scale (SACS), Children’s Global Assessment Scale (C-GAS), along- side consumer feedback questionnaires. | Reported significant improvements in global social and psychiatric functioning measured by C-GAS (*p* < .001); reduced risk of clinically significant mental health concerns measured by SDQ (*p* < .001); and reductions in the use and impact of drugs/alcohol measured by SACS (*p* < .001).  Participants and their families/wha ̄nau reported that the interventions were safe and appropriate, with perceived increased skill development around coping and communication. | Strong |
| **DeJong (2006)**  **Journal paper** | Inter-tribal residential boarding school as Therapeutic Residential Model site | United States  First Nations students (grades 1-8) | Approximately 200 students enrolled annually | Quantitative multiple time series design. | **Outcome Measures:** Retention and return rates; School bonding; Peer and social bonding; Adaptability and stress management; Meaning and identity; and Academic achievement  **Measurement**  **Instruments:** The Prevention and Planning Survey section of the Amercian Drug and Alcohol Survey (ADAS); Youth Risk Behaviour Survey; Jessor Alienation Inventory; BarOn Emotional Quotient Inventory; and, administration data. | There were no improvements in the outcome measures of school bonding, peer and social bonding, adaptability and stress management, meaning and identity; and academic achievement.  There were concerns raised in the initial evaluation regarding the low retention rate, concerns of over staffing, aspects of the environment that appeared to be detrimental to social development and emotional stability, and an unusually high proportion of students receiving psychiatric diagnosis and medication. The site was asked to address these issues, however retention rates remained low at this site throughout the course of funding, and there were a high number of assaults and psychiatric hospitalisations. | Weak |
| **Dobia (2014)**  **Report** | Aboriginal Girls Circle (AGC) program aimed to increase social connection, participation and confidence | Australia  Aboriginal girls attending secondary schools | 41 students participating in the AGC.  46 people were interviewed in stage 1, including: AGC girls = 16; Aboriginal staff = 7; non-Aboriginal staff = 7; parents = 8; community members = 8.  Survey participants in stage 2 included: Aboriginal students (n=41), non-Aboriginal students (n=16), and staff members (n=22) from various schools. | First stage **qualitative**: Field observations, and interviews and focus groups involving AGC participants, group leaders, community Elders and teachers.  Second stage **quantitative** pilot study**:** Student survey and teacher survey using a combination of pre-existing and purpose-designed measurement scales. | **Outcome Measures:** Resilience; Connectedness; Self-concept; Cultural identity  **Measurement**  **Instruments:** California Healthy Kids Survey (CHKS) Resilient Youth Development Module (RYDM) and RYDM Environmental Resiliency Scale; Self-Description Questionnaire (SDQ); and a cultural identity measure. | Reported outcomes included: increase in confidence, self-esteem, social skills and leadership ability; reports of more positive attitudes and improved capacity to take a more considered approach to conflict suggested greater resilience; and, reports of an increased sense of connection between participants, and a greater involvement with the school and school staff. Both students and Aboriginal staff highlighted the value of the girls coming together in shared acknowledgement of their cultural identity. | Weak |
| **Fleming (2012)**  **Journal paper** | SPARX computerized Cognitive Behavioral Therapy (cCBT) programme | New Zealand  Students excluded or alienated from mainstream education experiencing depression | 32 adolescents (34% Maori, 38% Pacific Island, 56% male) aged 13–16 | Immediate vs. delayed intervention randomized controlled trial. | **Outcome Measures:** Depression; hopelessness; locus of control; anxiety; and, quality of life.  **Measurement**  **Instruments:** The Child Depression Rating Scale Revised (CDRS-R); Reynolds Adolescent Depression Scale (RADS-2); Paediatric Quality of Life Enjoyment and Satisfaction Questionnaire (PQ-LES-Q); Spence Anxiety Scale; Kazdin Hopelessness Scale (HPLS); and the Children’s Nowicki- Strickland Internal-External Control Scale short (20 item) form (CNSIE). | Significant differences were found between cCBT and wait groups on the CDRS-R (baseline to 5-week mean change –14.7 versus –1.1, *p*<.001), remission (78% vs. 36%, *p* = .047) and on the Reynolds Adolescent Depression Scale (–4.6 vs. +3.2 *p* = .05) but not on other self-rating psychological functioning scales. In intent-to-treat analyses CDRS-R changes and remission remained significant. Gains were maintained at 10-week follow-up. However, there were no significant effects on measures of hopelessness, locus of control, anxiety, or quality of life. | Moderate |
| **Gray (1998)**  **Journal paper** | Health promotion program aimed at enhancing self-esteem and reducing drug use | Australia  Aboriginal students | 27 students pre-intervention; post-intervention 15 students in 1995 and 29 in 1996. | Mixed-methods | **Outcome Measures:** Patterns of drug consumption; attitudes to drug use; student self-esteem; and feeling about school  **Measurement**  **Instruments:** Self-report questionnaires completed by students, observational data and unstructured interviews with staff and community members | It was not possible to ascertain outcomes from questionnaire data due to the transient nature of the student population, the small number of participants, and the construction and administration of the questionnaire.  Qualitative data provided some indication of positive outcomes including: enhanced self-esteem; greater empowerment of female students; increased awareness of health and substance use issues; reinforcement of existing positive beliefs about health matters; reduced use of analgesics; and capacity development of staff and volunteers. | Weak |
| **Hall (2006)**  **Journal paper** | Inter-tribal residential boarding school as Therapeutic Residential Model site | United States  First Nations students (grades 1-8) | Over 250 students enrolled annually | Quantitative multiple time series design. | **Outcome Measures:** Retention and return rates; School bonding; Peer and social bonding; Adaptability and stress management; Meaning and identity; and Academic achievement  **Measurement**  **Instruments:** The Prevention and Planning Survey section of the Amercian Drug and Alcohol Survey (ADAS); Youth Risk Behaviour Survey; Jessor Alienation Inventory; BarOn Emotional Quotient Inventory; and, administration data. | Reported outcomes included: a reduction in behavioural incidents; a significant decrease in the amount of money spent on external mental health services; an increase in the retention rate; an increase in academic skills in selected areas; higher scores on pre-post measures of adjustment, interpersonal relationships and adaptability; and, reduced substance use over the school year | Weak |
| **LaFromboise (1995)** | Community-initiated, high school-based suicide prevention initiative, The Zuni Life Skills Development Program | United States  American Indian adolescents from the Zuni reservation in New Mexico | 128 high school students aged 14-19 (mean age 15.9) | Quasi-experimental study design: intervention and no-intervention non-randomised groups. | **Outcome Measures:** Suicide vulnerability; hopelessness; depression; and, self-efficacy  **Measurement**  **Instruments:** Suicide vulnerability, hopelessness, depression, and self-efficacy scales. Self-report survey, behavioural observation, and peer ratings. | Outcomes included:   - The intervention group was less suicidal *(M =* 54.34) than the no intervention group (M = 58.86), f(61) = 1.45, *p <* .07. - The intervention group showed significantly less feelings of hopelessness (M = 3.53) than the no-intervention group *(M* = 4.67), f(61) = 1.63, *p <* .05. - The intervention group was not less depressed (M = 3.29) when compared to the no-intervention group *(M* = 3.37), f(61) = .30, *ns*. - Students' self-efficacy ratings for skills covered in the curriculum showed no intervention effect. | Strong |
| **Lee (2008)**  **Journal paper** | A community-driven initiative to prevent substance misuse and increase respect for culture and elders | Australia  Young Aboriginal people in remote communities | 73 interviews with community members and other key stakeholders. Student interviews were also conducted. | **Qualitative:** interviews with community members, staff and stakeholders.  **Quantitative**: routine data from NT education department and police described school attendance and youth apprehensions. Data on substance use was used from a concurrent study. | **Outcome Measures:** School attendance; Youth apprehension rates; Levels of substance use  **Measurement**  **Instruments:** n/a | Reported outcomes included: Increased youth training and recreational opportunities; Increased communication between local agencies; Overall satisfaction with programme delivery  Comparing the 2 years before and after the Unit’s implementation, there were no significant changes in school attendance (55.9% versus 51.3%) or youth apprehensions (68 versus 75). | Moderate |
| **Lowe (2012)**  **Journal paper** | Innovative school- based cultural intervention targeting substance abuse - Cherokee Talking Circle (CTC) | U.S.A  Native American adolescent population | 179 Keetoowah- Cherokee high school students between 13 and 18 years of age who had been referred for substance abuse counseling and were enrolled in one of the participating high schools within the tribal jurisdictional area. Intervention condition (n = 92) control condition (n = 87). | A two-condition quasi-experimental study design comparing the Cherokee Talking Circle (CTC) culturally-based intervention condition (n = 92) with the Be A Winner Standard Education (SE) condition (n = 87). Data were collected at pre- intervention, immediate post-intervention, and 90-day post-intervention. | **Outcome Measures:** Self-reliance as measures by the Cherokee Self-Reliance Questionnaire; general life problems; internal and external behaviour; and, substance use problems.  **Measurement**  **Instruments:** Cherokee Self-Reliance Questionnaire, Global Assessment of Individual Needs - Quick (GAIN-Q) which includes four major scales – General Life Problem Index (GLPI), Internal Behavior Scale (IBS), External Behavior Scale (EBS), and Substance Problem Scale (SPS). | Significant improvements were found among all measurement outcomes for the CTC culturally- based intervention. These improvements were found post-intervention and increased on all measures at 3-month follow-up, suggesting that Improvements in Cherokee self-reliance, overall health problem scores, and substance problem scores for intervention group compared to the control group. | Moderate |
| **Miller (2011)**  **Journal paper** | A culturally adapted cognitive behavioural program for Aboriginal students | Canada  Aboriginal children aged 10-12 (grades 4-6) | Students (*n=* 533) including 192 Aboriginal students (mean age 9.77 years) | Cluster randomized control study: Anxiety levels in children were assessed across three time points. The active treatment group received the intervention in phase one and phase two (across all three time points), and the waitlist condition received the intervention in phase two (between time point 2 and 3). | **Outcome Measures:** Anxiety symptoms in children.  **Measurement**  **Instruments:** Multidimensional Anxiety Scale for Children (MASC) | Overall anxiety scores gradually decreased across the three time points for participants with elevated anxiety in both the active treatment and waitlist control groups. Students with initial elevated anxiety were the only group who experienced a significant change in overall anxiety from Time 1 to Time 2 and Time 2 to Time 3.  In Phase 1 the change in anxiety for the students in the active treatment condition was not significantly different from that of the waitlist condition. In Phase 2 there was no significant difference in anxiety levels comparing the group who received the intervention for 6 months with those who received the intervention for 3 months.  Children of Aboriginal background did not differ from non-Aboriginal children in anxiety levels, symptom decline, or in positive response to the program. | Strong |
| **Osborn (2013)**  **Journal paper** | A collaborative, community capacity building approach to re-contextualise Mental Health and Wellbeing (MHWB) understanding and curriculum to be taught in a very remote Aboriginal school (Mind Matters) | Australia  Anangu (Aboriginal) community educators | n/a | Key informant interviews (in person and written response) with Mind Matters staff, Anangu and non-Anangu remote educators involved in the program.  Base-line and follow-up mixed-method survey of student and community perceptions of the MHWB of young people. | **Outcome Measures:** The perceived strengths and benefits of this approach from the perspectives of Anangu educators  **Measurement**  **Instruments:** n/a | Reported outcomes included:   - The development of local understanding and language to discuss MHWB - An increased confidence to engage in conversations about MHWB with students and school community members. - Sense of achievement and building of confidence and capacity of Anangu educators - Development of Mind Matters resources in Pitjantjatjara language, and the production of posters and bilingual guides. - Anecdotal reports of a significant reduction in student violence. | Moderate |
| **Patten (2012)** | Targeted tobacco cessation intervention developed for Alaska Native youth | U.S.A.  Alaska Native youth | The first pilot enrolled nine adolescents (all female) aged 13–16 years; all nine attended the intervention program and 78% (7/9) completed follow-up. The second pilot enrolled 12 adolescents (eight females, four males) aged 12–17 years, of which seven attended the intervention program. Six of these seven participants (86%) completed follow-up. | Pilot study evaluated the feasibility and acceptability  Two intervention pilots were conducted from October 2010 to January 2011 using a non-randomized, uncontrolled study design with assessments at baseline and six-week follow-up. | n/a | In both pilots, participants rated the intervention as highly acceptable. A targeted cessation intervention was feasible and acceptable to AN youth. High rates of self-reported tobacco abstinence in one group. | Weak |
| **Spears (2006)**  **Journal paper** | A peripheral dormitory for First Nations children attending local schools, operating as a Therapeutic Residential Model site | United States  First Nations children in grades 1-12 | 185 boys and girls stay at the dormitory | Quantitative multiple time series design. | **Outcome Measures:** Retention and return rates; school bonding; peer and social bonding; adaptability and stress management; meaning and identity; and academic achievement.  **Measurement**  **Instruments:** The Prevention and Planning Survey section of the Amercian Drug and Alcohol Survey (ADAS); Youth Risk Behaviour Survey; Jessor Alienation Inventory; BarOn Emotional Quotient Inventory; and, administration data. | - Student retention increased from 40.7% in 2000-2001 to 68.4% in 2004-2005 - 75 students graduated in the four Therapeutic Residential Model years compared with 41 in the preceding 4 years. - Academic proficiency and ACT scores improved significantly - Cigarette use dropped significantly - There was an increase in feelings of inclusion and a decrease in lack of meaning - Behavioural incidents declined - Attitudes towards school and feeling of being liked changed dramatically when students attended the on-site school - Increased acceptance of mental health support by students - A decrease in “cutting” | Weak |
| **Tsey (2005)**  **Journal paper** | A school-based personal development and empowerment program | Australia  Indigenous students living in remote communities. | 50 students (9-12 years, grades 5-7) in one school and 20 students (grade 7) participated in the program.  One quarter of the student participants, and 8 staff members, were interviewed for evaluation. | Qualitative:  Semi-structured interviews | **Outcome Measures:** The extent to which participating in the program resulted in  personal empowerment, including enhanced analytical and  problem-solving skills, empathy, self identity, increased  participation in school activities, and decreased bullying, teasing and fighting among students  **Measurement**  **Instruments:** n/a | Reported outcomes include:   - Increased analytical and reflective skills - Greater ability to think for oneself and set goals - Less teasing and bullying in the school environment - An enhanced sense of identity, friendship and ´social relatedness´. | Strong |
| **Woods (2011)** | School-based early intervention program (the Kiwi ACE program) with Maori and Pacific adolescents experiencing depressive symptoms | New Zealand  Maori and Pacific students experiencing depressive symptoms | 24 Maori and Pacific students (average age 14 years) | Randomised control trial | **Outcome Measures:** Depression; changes in coping skills  **Measurement**  **Instruments:** Children’s Depression Inventory (CDI) | At immediate posttest (p = .045) and at one-year follow-up (p < .001) a significant effect for condition was obtained: the intervention group reported lower depressive symptoms. Efficacy of the intervention was supported by qualitative data obtained from focus groups. The majority of students identified positive changes in their coping strategies. | Moderate |
